# Enhanced photothermal heating and combination therapy of gold nanoparticles on a breast cell model

**DOI:** 10.1186/s13065-022-00859-1

**Published:** 2022-09-07

**Authors:** Amna H. Faid, Samia A. Shouman, Yehia A. Badr, Marwa Sharaky

**Affiliations:** 1 National Institute of Laser Enhanced Science (NILES), Cairo University, Giza, Egypt; 2 Pharmacology Unit, Cancer Biology Department, National Cancer Institute (NCI), Cairo University, Giza, Egypt

**Keywords:** Gold Nanoparticles, Drug delivery, Doxorubicin, Photothermal therapy, Cancer, Breast cell line, FTIR spectroscopy

## Abstract

Multi-drug resistance (MDR) in addition to the damage to non-malignant normal cells are the most difficult in cancer treatment. Drug delivery and Plasmonic photothermal therapy based on the use of resonant metallic nanoparticles have developed as promising techniques to destroy cancer cells selectively. In the present work, gold nanoparticles (AuNPs) were synthesized using trisodium citrate. The prepared AuNPs have a small size of 14 ± 4 nm and exhibit high stability with Zeta potential − 18 mV, AuNPs showed higher photothermal heating efficiency compared to irradiation with a 532 nm laser alone on the breast cancer cell line (MCF-7). Treatment of MCF-7 cells with 0.125 mM AuNPs coupled with laser irradiation for 6 min was found to significantly reduce (34%) the cell viability compared to 5% obtained with AuNPs in the same concentration and 26% with laser irradiation for 6 min without AuNPs. Moreover, the prepared AuNPs were used as an anticancer drug carrier for Doxorubicin (Dox), upon loading Dox to AuNPs there was a slight increase in the particle size to 16 ± 2 nm, FT-IR spectroscopic results showing the binding of Dox to AuNPs was through the –NH group. The potential cytotoxicity of the DOX@AuNPs nanocomposite was significantly increased compared to free DOX on the MCF7 cell line with a decrease in IC_50_. All these results suggested the potential use of AuNPs as therapeutic photothermal agents and drug carriers in cancer therapy.

**Supplementary Information:**

The online version contains supplementary material available at 10.1186/s13065-022-00859-1.

## Introduction

Cancer remains one of the major causes of death despite recent advancements in cancer treatment [1]. In 2020, 2.7 million people in the European Union were diagnosed with the disease, and another 1.3 million people lost their lives to it [2]. The world invests tens of billions of dollars and huge human capital every year in the development of new anti-tumor drugs. A major problem with conventional anticancer drugs involves their toxicity, which can lead to the death of healthy cells and cancer cells [3]. Recently, the emergence of nanotechnology had a significant impact on clinical treatment [4]. Photothermal therapy has developed as an alternative approach to non-invasive cancer therapy, which employs heat generated from the conversion of photon energy, for the localized destruction of cancer cells [5]. Heating sources such as near-infrared (NIR) or visible lasers are commonly used in conjunction with a photothermal agent to increase the temperature at the targeted site for killing the cancer cell [6]. Cancer cells are usually found to be more sensitive to heat than normal cells due to the more hypoxic, acidic, and nutrient-deficient microenvironment of the tumour. [7]. In comparison with other metal nanoparticles, noble metal (Cu, Hg, Ag, Pt, and Au) nanoparticles have increasingly attracted the attention of researchers. Among these, gold nanoparticles (AuNPs) are known to be the most stable and have been prepared with various shapes and structures. AuNPs are widely employed across the medical field owing to their excellent biocompatibility and high chemical and physical stability [8]. Recently, using noble metal nanoparticles as a photothermal agent and as a drug carrier has received much attention because of their special characteristic, especially the ultra-small size and the unique plasmonic characteristics in addition to the enhanced permeability and retention (EPR) effect of tumor tissues [9]. AuNPs have been used as unique drug delivery carriers in clinical applications due to their characteristics such as shape, size, and surface dependence [10]. AuNPs have many benefits that make them appropriate for photothermal cancer treatment as they can be administered into the local tumor area while minimizing non-specific distribution, and can be activated via laser light, creating the ability to penetrate into biological tissues, in addition, they can be modulated to create multifaceted cancer PTT and drug delivery systems [11]. Gold nanospheres (AuNS) were popularized by their ease of fabrication, small size, fast synthesis, and ease of ligand conjugation, making them attractive for PTT applications.^[12]^Despite having multiple benefits, AuNPs have some limitations, which limit their applications in medicines. The first is the toxicity of AuNPs without any capping or biocompatible layer, which is a significant concern. The toxicity of AuNPs depends on numerous parameters, such as composition, shape, size, coating, charge, hydrophobicity, solubility, and reactivity. The different biological environment also influences the toxicity encounters AuNPs for instance biofluids, intracellular media, and insertion in biovesicles. The second is the evolution of their biological medium properties. Studies indicate that AuNPs tend to aggregate within the lysosome, which modifies their optical properties and alters their activation under radiation. Thus, the AuNPs need an additional organic or biologic surface coating to make them stable. All these constraints limit the approval of AuNPs for clinical trials and commercial use [12] Among various chemotherapy drugs, doxorubicin (DOX) is a drug widely used as an anti-cancer drug. It kills cancer cells by inhibiting the synthesis of nucleic acid in the cell, To overcome the non-specificity and high toxicity of doxorubicin many researchers have proposed conjugation to nanoparticulate delivery systems to reduce the toxicity level while sustaining the therapeutic efficacy [13].

In this work, we employed the preparation and characterization of citrate capped AuNPs and their use as a photothermal agent in photothermal therapy on breast cancer (MCF-7) cells. subsequently preparation of DOX@AuNPs nanocomposite by modification of AuNPs surface with DOX as direct interaction of DOX with AuNPs. The progress in the formation of the new composite has been studied via UV–visible spectroscopy, TEM, and FTIRs. The obtained results indicate that the DOX@AuNPs produced a highly toxic effect on MCF7 compared to the effects of equal doses of free DOX so reducing the side effects and increasing the therapeutic efficiency.

## Materials and methods

### Chemicals

Tetrachlorauric acid (HAuCl_4_.3H_2_O), Trisodium Citrate salt, Dimethylsulphoxide (DMSO),RPMI-1640 medium,Sodium bicarbonate,Trypan blue, Fetal Bovine Serum, Penicillin/Streptomycin,Trypsin,Acetic acid, Sulphorhodamine-B (SRB),Trichloroacetic acid (TCA), Tris base 10 mM (PH 10.5) are obtained from Sigma Aldrich Chemical Co., St.Louis, Mo, U.S.A.

### Preparation of AuNPs

AuNPs were synthesized according to the standard wet chemical method [14, 15]. Trisodium citrate (38.8 mM, 10 mL) was added to a boiling HAuCl4 solution (1 mM, 100 mL). In addition, the yellow-colored solution of gold chloride turned the wine red in color. The formed particle size and shape were investigated using a TEM and UV–Visible spectrophotometer. In order to make sure that the cytotoxic effect is due to a laser-induced modification to AuNPs rather than other influences from the surrounding medium, absorption spectra were measured on AuNPs in culture media RPMI containing fetal bovine serum. The photostability of 1mMAuNPs has been studied by irradiation with DPSS (Diod Pumped Solid State) laser 532 nm and 250mW. The absorption spectra of the solution have been measured before exposure, and then it is irradiated with a laser source for 2 min, 4 min, 6 min, 8 min and10min. The absorption spectra have been measured after irradiation to monitor any change in the absorption spectra.

### Preparation of Dox@AuNPs nanocomposite

Dox-AuNPs nanocomposite was made according to *Vaithilingam* method [16]. 1 ml of different concentrations of Dox (10, 20, 30, 40 μM) were mixed drop-wise with 1 ml of 0.125 mM of AuNPs with continues stirring and sonicate for 10 min until deep red becomes blue.

### Characterization of AuNPs and Dox@AuNPs nanocomposite

The prepared AuNPs and Dox@AuNPs nanocomposite were characterized by UV–visible absorbance spectra using a double beam spectrophotometer *(PG instrument, T80*^+^*, UK.)*. 200 μl from AuNPs and Dox@AuNPs nanocomposite were diluted to 2 ml with distilled water then placed in 1 cm UV-quartz and the absorption was recorded within the appropriate scan range (200 to 800 nm). The spectra were taken against distilled water as a pure solvent reference for each sample. The morphology of the prepared AuNPs and Dox@AuNPs nanocomposite carried out using TEM—Nanotechnology& Advanced Material Central Lab. (NAMCL), Agriculture Research Center (ARC). Company name: FEI, Netherland. Model: Tecnai G20, Super twin, double tilt, and Applied voltage: 200 kV, Magnification Range: up to 1,000,000 X and Gun type: LaB6 Gun. A drop from very dilute solutions were deposited on an amorphous carbon-coated copper grid and left to evaporate at room temperature forming a monolayer then detected by TEM.IR measurements were carried out using FT-IR spectrometer (Shimadzu FT-IR 8400) in the range (500–4500 cm^−1^). Prepared samples (free DOX and DOX-AuNPs) were dried using a *lyophilizer*.IR spectra of powdered samples were diluted with a potassium bromide (KBr) pellet. Where 4–8 mg from the dried AuNPs were added to 200 mg KBr then careful grinding of the sample was of great importance for the elimination of errors caused by scattering. then the particle size and surface charges of the prepared AuNPs were analyzed through DLS with Zeta sizer 300 HAS (Malvern Instruments, Malvern, UK) based on photon correlation spectroscopy. Analysis time was 60 s and the average zeta potential was determined. The zeta potential of nanoparticulate dispersion was determined as such without dilution.

### Photothermal therapy on breast cell line

This method was carried out according to that of Skehan et al. (1990) [17, 18]. Cells were incubated with different concentrations of AuNPs (0.125, 0.25, 0.375, 0.5 mM) and then completed to a total of 200 μl volume/well using a fresh medium, and incubation was continued for 24 h. Cells was were obtained from the American Type Culture Collection (ATCC, Minnesota, USA). The tumor cell line maintained at National Cancer Institute (NCI), Cairo, Egypt. For each drug concentration, three wells were used. For the laser irradiation experiment, the DPSS (Diod Pumped Solid State laser) with wavelengths 532 nm and 250 mW was chosen (due to wavelength overlapping with the absorption band of the AuNPs). The microplate was divided as follows: Cells were seeded in 96-well microtiter plates at a concentration of 5 × 10^3^ cell /well in a fresh medium and left to attach to the plates for 24 h. The prepared 0.125 mM AuNPs were added leaving definite numbers of wells as control. The cells were left to grow overnight in the presence of AuNPs. In this way, the nanoparticles are incorporated inside cells by the endocytosis process. Cells were incubated with 1 mM AuNPs and then exposed to laser light at 250 mW for different times: 2, 4, 6, 8, and 10 min. Cells without nanoparticles have been exposed to laser light at 250 mW for the same different times to test the photothermal stability of the cell themselves. Following 24 h treatments, the cells were fixed with 50 μl cold 50% trichloroacetic acid for 1 h at 4 °C. Wells were washed 5 times with distilled water and stained for 30 min at room temperature with 50 μl 0.4% SRB dissolved in 1% acetic acid. The wells were then washed 4 times with 1% acetic acid. The plates were air-dried and the dye was solubilized with 100 μl/well of 10 mM tris base (ph 10.5) for 5 min on a shaker (Orbital shaker OS 20, Boeco, Germany) at 1600 rpm. To test for the potential cytotoxicity of Dox and Dox@AuNPs on the MCF7 cell line, cells were incubated with different concentrations of free DOX ((10, 20, 30, and 40 µM)) and Dox@AuNPs nanocomposite with the same concentrations then completed to a total of 200 μl volume/well using fresh medium and incubation was continued for 48 h. For each drug concentration, three wells were used. Following 48 h treatment, the cells were treated as previously described. The optical density (O.D.) of each well was measured spectrophotometrically at 564 nm with an ELIZA microplate reader (Meter tech. Σ 960, U.S.A.). The mean background absorbance was automatically subtracted and means values of each nano concentration were calculated. The percentage of cell survival was calculated as follows:

$$ {\text{Survival fraction }} = {\text{ O}}.{\text{D}}. \, \left( {\text{treated cells}} \right)/{\text{ O}}.{\text{D}}. \, \left( {\text{control cells}} \right). $$

The IC50 values (the concentrations of thymoquinone required to produce 50% inhibition of cell growth) “the experiment was repeated 3 times for each cell line.”.

### Statistical analysis

Data are expressed as the arithmetic mean ± SD. Statistical analysis was carried out using GraphPad Software Prism v5 (San Diego, USA). The statistical analysis of the transfection assay data was done using Tukey multiple comparison test with a single pooled variance. Differences were considered statistically significant when p ≤ 0.05.

## Results and discussion

**Fig. 1:**
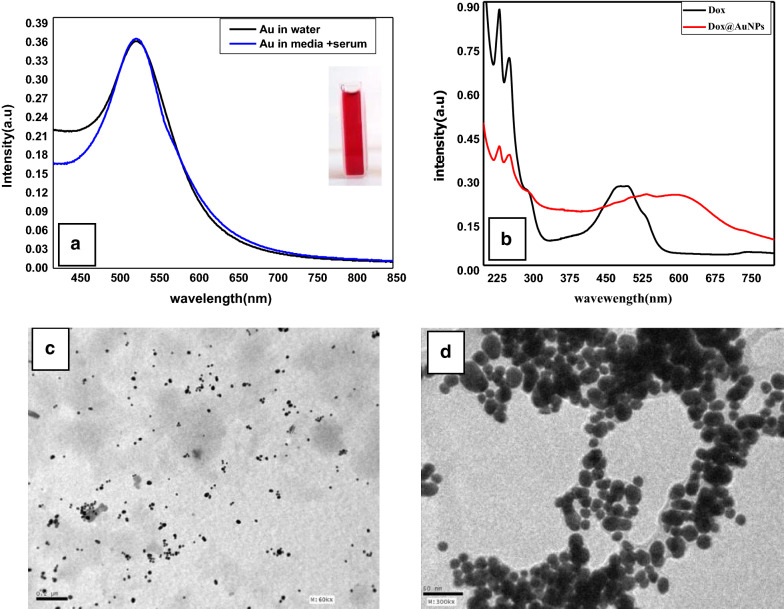
UV- Visible Spectrum of **a** AuNPs with a digital photograph and AuNPs in media and **b** Dox, Dox@AuNPs nanocomposite and TEM images of **c** AuNPs and **d** Dox @AuNPs nanocomposite

AuNPs were synthesized by the chemical reduction of HAuCl_4_ using trisodium citrate as a reducing and capping agent. The formation of ruby-colored dispersion was an indicator of nanoparticles formation of gold nanoparticles [19]. As shown in Fig. 1a, the prepared AuNPs show absorption in the visible range due to Surface Plasmon Resonance (SPR) at 520 nm which indicates the stable state of the AuNPs clearly. The narrow band of the curve corresponds mostly to the monodisperse nature of AuNPs, without any aggregation and agglomeration. TEM image was employed to clarify the morphology of formed AuNPs, the particles are spherical with approximate size (14 ± 4 nm) with uniform size distribution Fig. 1c. As shown in Fig. 1a, there is no obvious change in AuNPs absorption peak upon addition AuNPs to the culture media which indicate the stability of AuNPs in culture media this may be due to the presence of serum in the medium, particles, in this case, were suggested to be coated with serum where the surface of AuNPs was modified by non-specific adsorption of serum proteins [20].

The UV–visible absorption of Dox shows one band in the visible range extends from 400 nm to about 560 nm. This band is characterized by anthracyclines. Dox absorbs also in the ultraviolet range as it has absorption peaks from 200 to 300 nm [21]. Adding colloidal AuNPs to Dox, the band intensity decreased. This decrease is accompanied by the appearance of a new band at 630 nm Fig. 1b. The appearance of this new band is a consequence of aggregation which is due to interparticle interaction between the adjacent AuNPs upon the addition of drugs. The aggregation can be verified by a slight increase in the AuNPs size as shown in TEM image Fig. 1d. The aggregation of AuNPs might be caused by the replacement of citrate molecules with Dox leading to the formation of the Dox@AuNPs complex. In addition, the greater electrostatic attraction of active groups in Dox with AuNPs than citrate groups might be responsible for the additional band at higher wavelengths. It has been shown theoretically and experimentally that aggregation of AuNPs leads to another plasmon absorption at longer wavelengths when the individual nanoparticles are electronically coupled to each other [22]. TEM images of Dox@AuNPs nanocomposite Fig. 1d; reveal that the nanocomposite possessed a regularly spherical shape and smooth surface with slight increases in the particle size to 16 ± 2 nm. Here the phenomenon of the plasmon coupling appeared clearly with linearly arranged coupled particles.

**Fig. 2:**
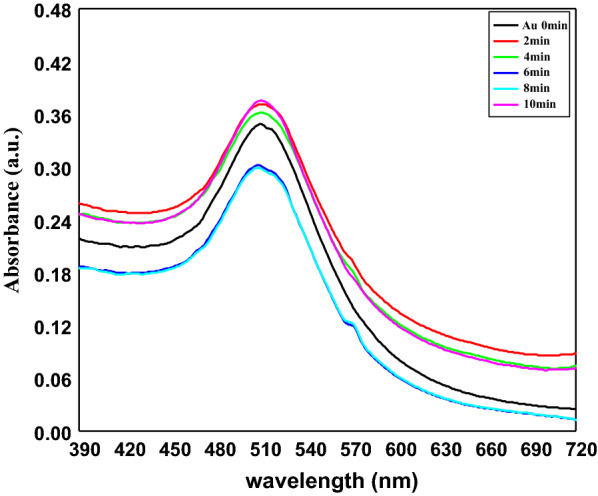
Effect of DPSS laser irradiation on AuNPs

The significance of the need for the photostability of AuNPs comes from the utilization of AuNPs in the treatment of cancer. When these particles are decided to be used as hyperthermic agents in photothermal therapy, the target tissue or cells will be exposed to laser light. We have to test the stability of AuNPs by exposure to the same laser light for the same exposure time. As shown in Fig. 2, the irradiation of AuNPs using DPSS (Diod Pumped Solid State) laser for different times has no effect on the Surface Plasmon Resonance (SPR) of the particles which indicates the photothermal stability of AuNPs.

**Fig. 3:**
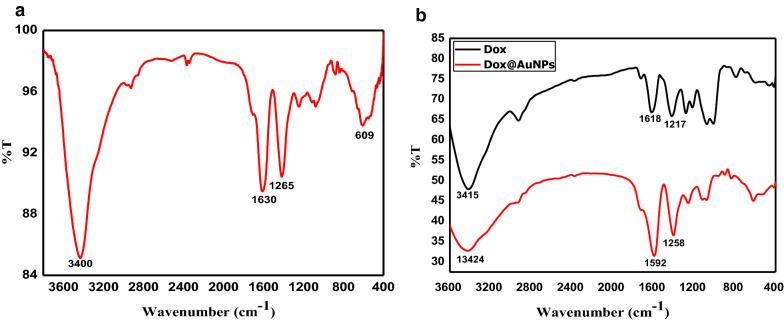
FTIR spectra of **a** AuNPs and **b** Dox and Dox@AuNPs nanocomposite

The prepared AuNPs were further characterized via FTIRs Fig. 3a, the recorded spectra of citrate capped AuNPs showed two main peaks at 3400 and 1630 cm^−1^ and a medium peak at 1265 cm^−1^. The broad peak at 3400 cm^−1^ correlates with O–H stretching vibrations due to intermolecular hydrogen bonding. Moreover, the peak at 609 cm^−1^ corresponded to C=O stretching. While peaks at 1630 and 1265 cm^−1^ correspond to –C=O stretching and C-O stretching respectively. FTIRs analysis confirmed that the surface of the colloidal AuNPs was covered with citrate groups, which are important for the stability of the prepared nanoparticles [23]. The binding interaction of Dox@AuNPs was further investigated using FT-IR studies. The IR spectra of free Dox and Dox@AuNPs are shown in Fig. 3b. Dox shows bands at 3415 cm^−1^, 1217 cm^−1^ and 1618 cm^−1^ corresponding to–NH stretching frequency, C-N stretching and NH_2_ bending respectively. In the case of Dox@AuNPs, there were a decrease in intensity of all bands and NH which is now broadened and slightly shifted to higher wavelengths at 3424 cm^−1^. And there is a redshift for C-N stretching to 1258 cm^−1^ and blue shifts for NH_2_ bending to1592 cm^−1^ respectively Fig. 5B. It could be understood that it is the free –NH group that is likely to be involved in the binding of Dox on AuNPs surface as it is well known that gold has a strong affinity towards amino groups [24]. The results given here were accorded well with Hua Qin Yin et al*.* [25].

**Fig. 4:**
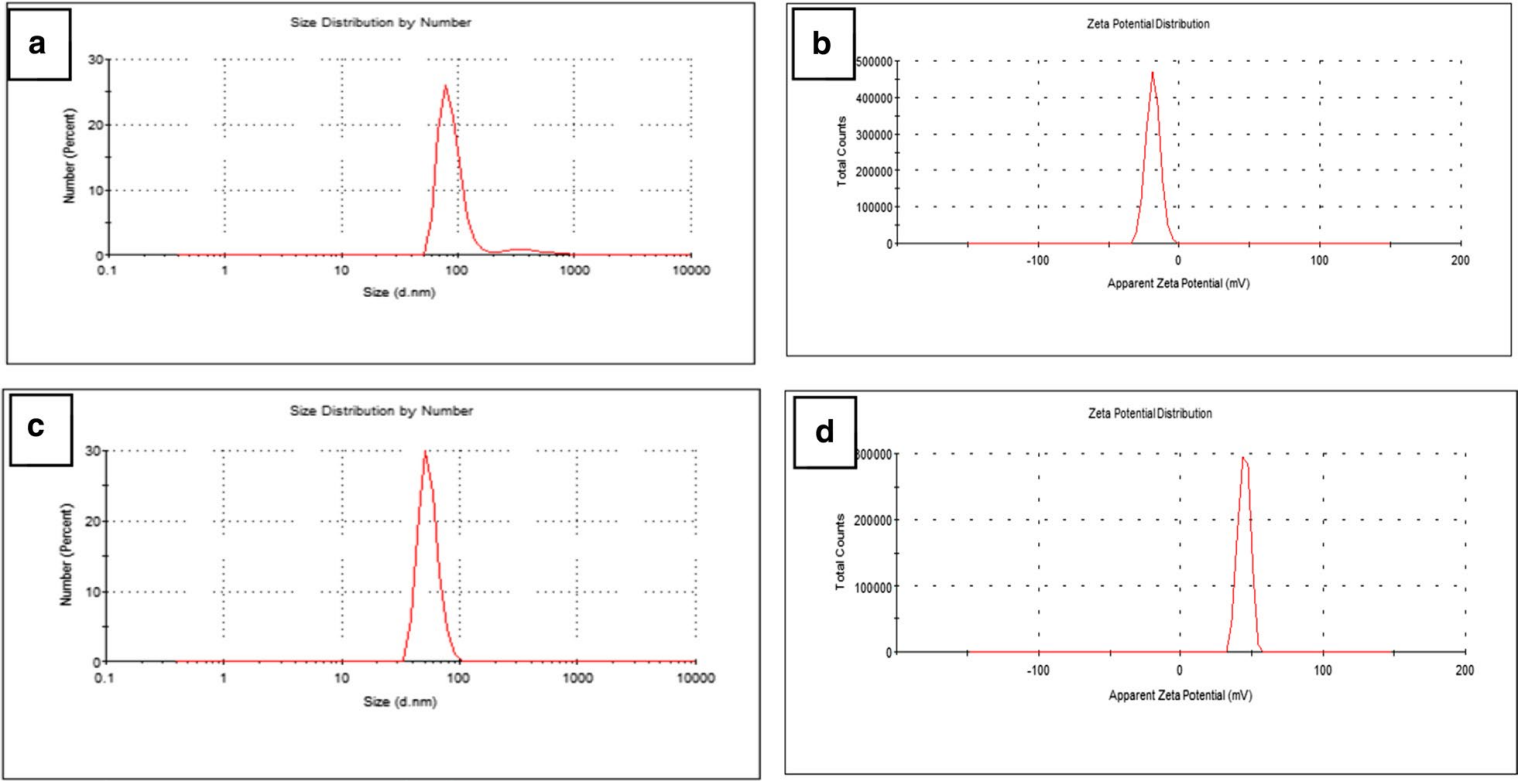
**a**, **b** Size distribution and zeta potential of AuNPs and **c**, **d** Size distribution and zeta potential of Dox@AuNPs

DLS and zeta potential measurements were carried out to study the hydrodynamic diameter and surface charge of the synthesized colloidal AuNPs. Typically, gold nanoparticles with zeta potential greater than + 20 mV or less than − 20 mV have sufficient electrostatic repulsion between neighboring correspondingly charged particles to remain stable. The results particle size and zeta potential of AuNPs were 105 nm and − 18 mV respectively as shown in Fig. 4a, b. the hydrodynamic size is larger than that characterized by TEM, which can be attributed to a certain amount of hydrated molecules around the core of the water-soluble AuNPs. after loading Dox on AuNPs the particle size and zeta potential were shifted to Size 54 nm,44.4 mV as shown in Fig. 4c, d. Zeta potential is considered as an indicator of the stability of nanoparticles. This negative value of zeta potential is due to the presence of three deprotonated anionic carboxyl groups of citrate ions which verify the repulsive interaction between nanoparticles and aim to prevent the agglomeration of AuNPs [26, 27].

**Fig. 5:**
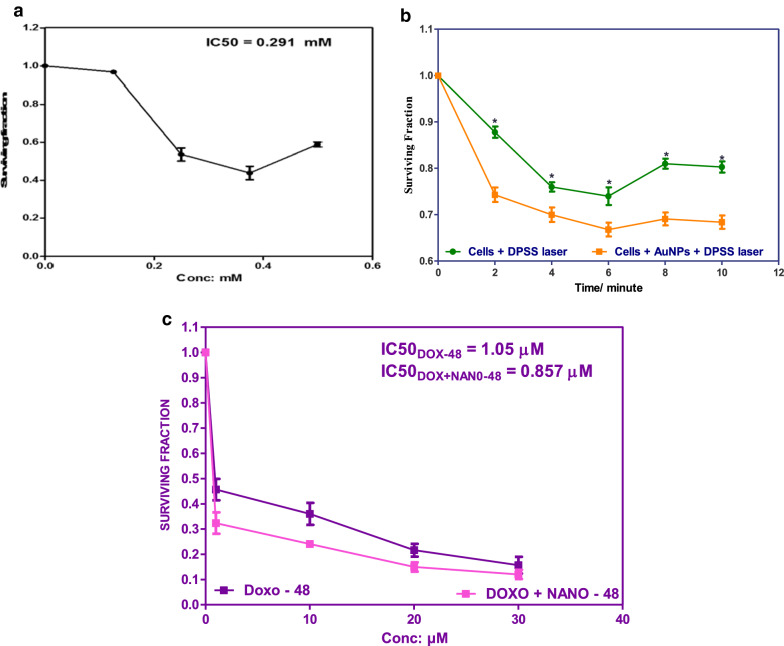
**a** Cytotoxicity of different AuNPs concentrations on MCF7, **b** effect of irradiation with DPSS laser at a different time on MCF7 cells viability and MCF7 incubated with 0.125 mM of AuNPs after 24 h, **c** Cytotoxicity of different Dox and Dox@AuNPs composite concentrations on MCF7

### Photothermal therapy and drug delivery of AuNPs on MCF7

In order to investigate the cytotoxicity of AuNPs on MCF-7 cells without irradiation, MCF-7 cells were treated with different concentrations of AuNPs (0.125, 0.250, 0.375,0.5 mM) for 24 h as shown in Fig. 5a, about 95% cell viability was noticed upon treatment with 0.125 mM AuNPs which indicates the safety of this concentration to be used in photothermal and drug delivery. Figure 5b illustrates the percentage of MCF-7 cell viability incubated with 0.125 mM of AuNPs and treatment with increasing times of irradiation (2 min, 4 min, 6 min, 8 min, and 10 min) with DPSS laser of 532 nm and 250 mW. Results showed a significant decrease indicated by13%, 24%, 26%, 19%, and 20% compared 26%, 30%, 34%, 31%, and 32% after laser irradiation in MCF7 cell and incubation with the AuNPs respectively as shown in Additional file 1: Table S1. As increasing the irradiation time led to a progressive increase in the temperature of the medium, the results showed a gradual decrease in cell survival by increasing the time of laser exposure on MCF-7 cell viability in the absence of AuNPs since cancer cells have been shown to be more susceptible to high temperature compared to normal cells [7]. The enhancement in the cytotoxicity of AuNPs upon laser irradiation is due to inducing localized hyperthermia resulting in the killing of MCF-7 cells. Such an increase in the local temperature might have resulted in the thermal denaturation of proteins, which may be the main factor in cell injury and cell death [28]. The reason can be attributed to being due to the strong surface plasmon absorption of AuNPs which offer great potential in photothermal therapeutic applications. Our results were in accordance with Sunil Kumar Surapaneni et al. [29]. It has been found that the strong absorbed radiation is converted efficiently into heat on a picosecond time domain due to electron–phonon and phonon–phonon processes. Thus, upon the laser irradiation at the surface plasmon absorption band, AuNPs absorb photon energy and then immediately transfer it into heat energy.Then the Nanoparticles lattice cools rapidly by exchanging energy with the surrounding medium on the timescale of ~ 100 ps [30]. The efficient photothermal killing of MCF-7 cells by AuNPs can be attributed to their smaller size. As reported in a previous study, smaller AuNPs have been shown to be more efficient in the photothermal killing of cancer cells [31]. Such an increase in the local temperature might have resulted in the thermal denaturation of proteins, which may be the main factor in cell injury and cell death [32].

Figure 5c, reveals the effect of different concentrations of Dox and Dox@AuNPs nanocomposite (10, 20, 30, and 40 µM) on the percentage of survival of MCF7 after 48 h. There was a concentration-dependent decrease in cellular proliferation compared to its respective control. Dox@AuNPs exhibited a lower *IC50* than free Dox. The cytotoxicity of Dox@AuNPs nanocomposite may be improved due to better accumulation of drug at its site of action due to targeted delivery. A possible explanation for the activity enhancement of Dox@AuNPs nanocomposite is the improvement due to the internalization of Dox@AuNPs nanocomposite by an endocytosis mechanism. which indicated that the targeted nanocarriers greatly increased cellular uptake of Dox by MCF7 cancer cells. Generally, nanoparticles are nonspecifically internalized into cells via endocytosis or phagocytosis compared to the passive diffusion mechanism of free Dox into cells [33]. As shown in Fig. 5a and c, free AuNPs showed almost non-cytotoxic at 0.125 mM, and the Dox@AuNPs caused a strong decrease in cell viability. This result is the same as our expectation, that the free gold nanocarrier has good biocompatibility, and it showed a good cytotoxicity effect after laser irradiation and loading with Dox [34].

## Conclusions

The present work demonstrated the preparation of small highly stable citrate-capped AuNPs with zeta potential − 18 mV. AuNPs exhibited a photothermal therapeutic effect on MCF-7 cells upon irradiation using laser intensity of 250 mW for 6 min compared to laser alone. Moreover, a method for loading Dox on AuNPs forming Dox@AuNPs nanocomposite for breast cancer treatment was demonstrated. The prepared AuNPs and Dox@AuNPs were spherically shaped with an average particle size of 14 ± 4 nm and 16 ± 2 nm respectively. The results showed that loading DOX on AuNPs significantly enhance the anti-proliferation activity on the MCF7 cell line. The cell viability reduced significantly compared to free Dox. Overall, these nanoparticle complexes could be proposed as potent drug delivery vehicles for cancer drugs such as DOX, as well as other drugs in the prospective studies.

## Supplementary Information

**Additional file 1: Table S1**. Statistical information for the effect of irradiation with DPSS laser at a different time on MCF7 cells viability and MCF7 incubated with 0.125mM of AuNPs after 24 hr.

## Data Availability

All data generated or analysed during this study are included in this published article [and its additional files].

## References

[1] McGuire S. World Cancer Report 2014. Geneva, Switzerland: World Health Organization, International Agency for Research on Cancer, WHO Press, 2015. Adv Nutr 2016; **7**(2): 418–9. 10.3945/an.116.012211 PMC4785485 26980827

[2] Commission, E., D.-G.f. Research, Innovation, et al. Cancer screening in the European Union 2022. Publications Office of the European Union.

[3] Rabiee N, Yaraki MT, Garakani SM (2020). Recent advances in porphyrin-based nanocomposites for effective targeted imaging and therapy. Biomaterials.

[4] Zhu J, Tang X, Jia Y (2020). Applications and delivery mechanisms of hyaluronic acid used for topical/transdermal delivery—A review. Int J Pharm.

[5] Faid AH, Shouman SA, Thabet NA (2022). Laser enhanced combinatorial chemo-photothermal therapy of green synthesis gold nanoparticles loaded with 6mercaptopurine on breast cancer model. J Pharm Innov.

[6] Macchi S, Jalihal A, Hooshmand N (2022). Enhanced photothermal heating and combination therapy of NIR dye via conversion to self-assembled ionic nanomaterials. J Mater Chem B.

[7] Abadeer NS, Murphy CJ (2016). Recent progress in cancer thermal therapy using gold nanoparticles. J Phys Chem C.

[8] Hu X, Zhang Y, Ding T (2020). Multifunctional gold nanoparticles: a novel nanomaterial for various medical applications and biological activities. Front Bioeng Biotechnol.

[9] Yang W, Xia B, Wang L (2021). Shape effects of gold nanoparticles in photothermal cancer therapy. Mater Today Sustain.

[10] Sriubas M, Bockute K, Palevicius P (2022). Antibacterial activity of silver and gold particles formed on titania thin films. Nanomaterials (Basel, Switzerland).

[11] Vines JB, Yoon JH, Ryu NE (2019). Gold nanoparticles for photothermal cancer therapy. Front Chem.

[12] Singh P, Mijakovic I (2021). Advances in gold nanoparticle technology as a tool for diagnostics and treatment of cancer. Expert Rev Mol Diagn.

[13] Schneeweiss A, Möbus V, Tesch H (2019). Intense dose-dense epirubicin, paclitaxel, cyclophosphamide versus weekly paclitaxel, liposomal doxorubicin (plus carboplatin in triple-negative breast cancer) for neoadjuvant treatment of high-risk early breast cancer (GeparOcto-GBG 84): a randomised phase III trial. Eur J Cancer.

[14] Turkevich J, Stevenson PC, Hillier J (1951). A study of the nucleation and growth processes in the synthesis of colloidal gold. Discuss Faraday Soc.

[15] Bajaj M, Wangoo N, Jain DVS (2020). Quantification of adsorbed and dangling citrate ions on gold nanoparticle surface using thermogravimetric analysis. Sci Rep.

[16] Deinavizadeh M, Kiasat A, Hooshmand N (2021). Smart NIR-light and pH responsive doxorubicin-loaded GNRs@SBA-15-SH nanocomposite for chemo-photothermal therapy of cancer. Nanophotonics.

[17] Skehan P, Storeng R, Scudiero D (1990). New colorimetric cytotoxicity assay for anticancer-drug screening. J Natl Cancer Inst.

[18] Ramadan MA, Sharaky M, Faid AH (2022). Ionic gelation synthesis, characterization and cytotoxic evaluation of chitosan nanoparticles on different types of human cancer cell models. Egypt J Chem.

[19] Dong J, Carpinone PL, Pyrgiotakis G (2020). Synthesis of precision gold nanoparticles using Turkevich method. Kona.

[20] Shimaa A, Hazem S, Mahmoud A (2012). Laser-induced modifications of gold nanoparticles and their cytotoxic effect. J Biomed Opt.

[21] Li S, Amat D, Peng Z (2016). Transferrin conjugated nontoxic carbon dots for doxorubicin delivery to target pediatric brain tumor cells. Nanoscale.

[22] Li LS, Ren B, Yang X (2021). Hyaluronic acid-modified and doxorubicin-loaded gold nanoparticles and evaluation of their bioactivity. Pharmaceuticals.

[23] Tavakkoli Yaraki M, Tan YN (2020). Recent advances in metallic nanobiosensors development: colorimetric, dynamic light scattering and fluorescence detection. Sens Int.

[24] Lodhi MS, Khan MT, Aftab S (2021). A novel formulation of theranostic nanomedicine for targeting drug delivery to gastrointestinal tract cancer. Cancer Nanotechnol.

[25] Yin HQ, Shao G, Gan F (2020). One-step, rapid and green synthesis of multifunctional gold nanoparticles for tumor-targeted imaging and therapy. Nanoscale Res Lett.

[26] Jelen Ž, Majerič P, Zadravec M (2021). Study of gold nanoparticles’ preparation through ultrasonic spray pyrolysis and lyophilisation for possible use as markers in LFIA tests. Nanotechnol Rev.

[27] Liang C, Cheong JY, Sitaru G (2022). Size-dependent catalytic behavior of gold nanoparticles. Adv Mater Interfaces.

[28] Yee Foo Y, Saw WS, Periasamy V (2019). Green synthesised-gold nanoparticles in photothermal therapy of breast cancer. Micro Nano Lett.

[29] Surapaneni SK, Bashir S, Tikoo K (2018). Gold nanoparticles-induced cytotoxicity in triple negative breast cancer involves different epigenetic alterations depending upon the surface charge. Sci Rep.

[30] Nasseri B, Turk M, Kosemehmetoglu K (2020). The pimpled gold nanosphere: a superior candidate for plasmonic photothermal therapy. Int J Nanomed.

[31] Pattani V, Shah J, Atalis A (2015). Role of apoptosis and necrosis in cell death induced by nanoparticle-mediated photothermal therapy. J Nanoparticle Res.

[32] Saw WS, Ujihara M, Chong WY (2018). Size-dependent effect of cystine/citric acid-capped confeito-like gold nanoparticles on cellular uptake and photothermal cancer therapy. Colloids Surf B Biointerfaces.

[33] Zhang C, Zhang F, Han M (2020). Co-delivery of 5-fluorodeoxyuridine and doxorubicin via gold nanoparticle equipped with affibody-DNA hybrid strands for targeted synergistic chemotherapy of HER2 overexpressing breast cancer. Sci Rep.

[34] Pakravan A, Azizi M, Rahimi F (2021). Comparative effect of thermo/pH-responsive polymer-coated gold nanocages and hollow nanostars on chemo-photothermal therapy of breast cancer cells. Cancer Nanotechnol.

