# Supplementary material for: Enhanced photothermal heating and combination therapy of gold nanoparticles on a breast cell model
Source: BMC Chem. 2022 Sep 7;16(1):66. doi: 10.1186/s13065-022-00859-1 (PMC9454161; doi:10.1186/s13065-022-00859-1)
Supplement: Supplementary file 1 — Additional file 1: Table S1. Statistical information for the effect of irradiation with DPSS laser at a different time on MCF7 cells viability and MCF7 incubated with 0.125mM of AuNPs after 24 hr. [file 13065_2022_859_MOESM1_ESM.docx]

**Enhanced Photothermal Heating and Combination Therapy of Gold Nanoparticles on a Breast Cell Model**

Amna H. Faid^a,^*, Samia A. Shouman^b^, Yehia A. Badr^a, ‡^, Marwa Sharaky^b^

^a^ National Institute of Laser Enhanced Science (NILES), Cairo University, Giza, Egypt.

^b^ National Cancer Institute (NCI), Cairo, Egypt.

*Corresponding author. E-mail address: [amna.hussein@cu.edu.eg](mailto:amna.hussein@cu.edu.eg)

Table(1): Statistical information for the effect of irradiation with DPSS laser at a different time on MCF7 cells viability and MCF7 incubated with 0.125mM of AuNPs after 24 hr.

| Source of Variation | P value summary | Significant? |  |  |
| --- | --- | --- | --- | --- |
| Interaction | *** | Yes |  |  |
| Column Factor | *** | Yes |  |  |
| Time | *** | Yes |  |  |
|  |  |  |  |  |
| Source of Variation | Df | Sum-of-squares | Mean square | F |
| Interaction | 5 | 0.03848 | 0.007696 | 46.72 |
| Column Factor | 1 | 0.1275 | 0.1275 | 774.0 |
| Time | 5 | 0.7109 | 0.1422 | 863.0 |
| Residual | 60 | 0.009885 | 0.0001647 |  |
|  |  |  |  |  |
| Number of missing values | 0 |  |  |  |
|  |  |  |  |  |
| Bonferroni posttests |  |  |  |  |
|  |  |  |  |  |
| Cells + DPSS laser vs Cells + AuNPs + DPSS laser |  |  |  |  |
| Time | Cells + DPSS laser | Cells + AuNPs + DPSS laser | Difference | 95% CI of diff. |
| 0.0000 | 1.000 | 1.000 | 0.0000 | -0.02022 to 0.02022 |
| 2.000 | 0.8780 | 0.7430 | -0.1350 | -0.1552 to -0.1148 |
| 4.000 | 0.7600 | 0.7000 | -0.0600 | -0.08022 to -0.03978 |
| 6.000 | 0.7400 | 0.6680 | -0.07200 | -0.09222 to -0.05178 |
| 8.000 | 0.8100 | 0.6910 | -0.1190 | -0.1392 to -0.09878 |
| 10.00 | 0.8030 | 0.6840 | -0.1190 | -0.1392 to -0.09878 |
|  |  |  |  |  |
| Time | Difference | t | P value | Summary |
| 0.0000 | 0.0000 | 0.0000 | P > 0.05 | ns |
| 2.000 | -0.1350 | 18.22 | P<0.001 | *** |
| 4.000 | -0.0600 | 8.097 | P<0.001 | *** |
| 6.000 | -0.07200 | 9.716 | P<0.001 | *** |
| 8.000 | -0.1190 | 16.06 | P<0.001 | *** |
| 10.00 | -0.1190 | 16.06 | P<0.001 | *** |
|  |  |  |  |  |
